# Evaluation of Residual Human-Induced Pluripotent Stem Cells in Human Chondrocytes by Cell Type-Specific Glycosphingolipid Glycome Analysis Based on the Aminolysis-SALSA Technique

**DOI:** 10.3390/ijms21010231

**Published:** 2019-12-28

**Authors:** Takuji Miyazaki, Hisatoshi Hanamatsu, Liang Xu, Tomohiro Onodera, Jun-ichi Furukawa, Kentaro Homan, Rikiya Baba, Toshisuke Kawasaki, Norimasa Iwasaki

**Affiliations:** 1 Department of Orthopedic Surgery, Hokkaido University Graduate School of Medicine, Kita 15, Nishi 7, Kita-ku, Sapporo, Hokkaido 060-8638, Japan; takuzimiyazaki@gmail.com (T.M.); xuliang811026@gmail.com (L.X.); k.houman@med.hokudai.ac.jp (K.H.); baba76767688@gmail.com (R.B.); niwasaki@med.hokudai.ac.jp (N.I.); 2 Department of Advanced Clinical Glycobiology, Faculty of Medicine and Graduate School of Medicine, Hokkaido, Kita 21, Nishi 11, Kita-ku, Sapporo, Hokkaido 001-0021, Japan; h_hanamatsu@med.hokudai.ac.jp; 3 Global Station for Soft Matter, Global Institution for Collaborative Research and Education (GSS, GI-CoRE), Hokkaido University, Kita 21, Nishi 11, Kita-ku, Sapporo, Hokkaido 001-0021, Japan; 4 Research Center for Glycobiotechnology, Ritsumeikan University, 1-1-1 Nojihigashi, Kusatsu, Shiga 525-8577, Japan; tkawasak@fc.ritsumei.ac.jp

**Keywords:** cartilage damage, osteoarthritis, iPSCs, chondrocytes, GSL-glycome analysis, aminolysis-SALSA, tumorigenicity, glycoconjugates

## Abstract

Cartilage damage may eventually lead to osteoarthritis because it is difficult to repair. Human-induced pluripotent stem cell (iPSC)-derived chondrocytes may potentially be used to treat cartilage damage, but the tumorigenicity of iPSCs is a major concern for their application in regenerative medicine. Many glycoconjugates serve as stem cell markers, and glycosphingolipids (GSLs) including H type 1 antigen (Fucα1-2Galβ1-3GlcNAc) have been expressed on the surface of iPSCs. The purpose of the present study was to investigate whether GSL-glycome analysis is useful for quality control of residual iPSCs in chondrocytes. We performed GSL-glycome analysis of undifferentiated iPSCs in chondrocytes by combining glycoblotting and aminolysis-sialic acid linkage-specific alkylamidation (SALSA) method, enabling the detection of small quantities of iPSC-specific GSL-glycans from 5 × 10^4^ cells. Furthermore, we estimated the residual amount of iPSCs using R-17F antibody, which possesses cytotoxic activity toward iPSCs that is dependent on the Lacto-*N*-fucopentaose I (LNFP I) of GSL. Moreover, we could detect a small number of LNFP I during mesenchymal stem cells (MSCs) differentiation from iPSCs. This is the first demonstration that GSL-glycome analysis is useful for detecting undifferentiated iPSCs, and can thereby support safe regenerative medicine.

## 1. Introduction

Since articular cartilage has poor self-healing ability due to its specific structure, damaged cartilage can eventually lead to osteoarthritis. Autologous chondrocyte implantation (ACI) has been reported as a regenerative medicine for cartilage repair [1]. ACI has advantages over existing methods in which the injured part is covered with hyaline cartilage, but there remain issues such as the sacrifice of healthy cartilage, the need for two operations, and difficulty in acquiring a sufficient number of cells due to low proliferation ability. In addition, Roberts et al. reported that more than half of the cartilage tissue repaired with ACI were predominantly fibrocartilage composed of type I collagen and IIA procollagen, which were different from normal hyaline cartilage composed of Type II collagen [2].

Human-induced pluripotent stem cells (iPSCs) proliferate indefinitely in an undifferentiated state, and their pluripotency makes them capable of differentiating into any tissue in the human body [3]. Regenerative medicine using iPSC-derived cells has potential for repairing defective cartilage. Indeed, iPSCs can be differentiated into chondrogenic lineages [4], and iPSC-derived cartilaginous particles can repair articular cartilage defects in mini-pigs. However, the tumorigenicity and dedifferentiation of iPSCs are barriers that must be overcome before the true potential of iPSCs in regenerative medicine can be realized. Since even a small number of residual iPSCs may form teratomas [5], it is a prerequisite for clinical application that the final products contain no iPSCs. To evaluate whether iPSCs are included, fluorescence labeling and observation with a fluorescence microscope was applied [6], as has labeling and evaluation by flow cytometry [7], but it is difficult to confirm that all iPSCs are actually labeled using these techniques. Moreover, undifferentiated cell removal methods targeting iPS-specific glycans have been reported, such as elimination by cell sorting using an antibody recognizing SSEA-5 [8], and elimination by a recombinant lectin-toxin fusion protein [7]. Recently, Matsumoto et al. reported that R-17F was an antibody that detects undifferentiated iPSCs by recognizing the Lacto-*N*-fucopentaose I (LNFP I) of GSL, and consequently exerts cytotoxic activity [9]. However, staining (detection) for the monitoring and removal of all undifferentiated iPSCs following induction of differentiation is very difficult, and new methods that can overcome these problems are much needed.

The cellular surface is coated with a dense glycocalyx composed of glycoconjugates such as glycosphingolipids (GSLs) and glycoproteins, and many studies have attempted to identify cell-specific glycan markers. Undifferentiated iPSCs are also coated by unique glycoconjugates and GSLs such as SSEA-3, SSEA-4, Globo H, and H type 1 antigen, which serve as stem cell markers [10]. Expression of stem cell-specific globo and (n)lacto series GSLs is rapidly diminished upon differentiation, whereas expression of ganglioside GSLs such as GM3, GM2, and GD3 is increased [11]. Furthermore, we recently reported that expression of GM3, the most abundant ganglioside in chondrocytes, is gradually decreased during the process of chondrocytes hypertrophy [12]. The GSL-glycome on the cell surface is significantly altered upon differentiation and hypertrophy, and changes in GSL-glycans can determine cell fate.

Previously, we reported that qualitative and quantitative cellular glycomics of GSLs based on rhodococcal endoglycosylceramidase (EGCase)-assisted glycan cleavage, followed by glycan preparation by glycoblotting method [13]. Recently, we developed aminolysis-sialic acid linkage-specific alkylamidation (SALSA) method, which enable discrimination of sialic acid linkage isomers on GSL-glycans by mass spectrometric analysis [14]. Moreover, we showed that ganglioside GSLs containing α2,3-linked sialic acid residue could be derivatized with various amine analogues. We demonstrated that α2,3-sialylated GM1 could be derivatized with methyl- or ethyl-amine and observed with a mass difference of 14 Da. The ionization efficiency was not greatly changed after mixing methyl and ethyl derivatives in equal quantities. 

In the present study, we analyzed the GSL-glycome of co-cultured undifferentiated iPSCs and chondrocytes by glycoblotting-SALSA method. We focused on the R-17F antibody that binds selectively to iPSCs [9]. R-17F is a subclone obtained from hybridoma 17, which was obtained by intraperitoneally injecting iPSCs into mice and identifying the resulting hybridomas that exhibit reactivity toward surface antigens on iPSCs, but not toward the human EC cell line or the human fibroblast cell line. R-17F recognizes H type 1 glycans of GSLs (Fucα1-2Galβ1-3GlcNAc) that are specifically expressed in undifferentiated iPSCs, and displays dose-dependent cytotoxicity against iPSCs. Therefore, we hypothesized that it may be possible to confirm the iPSC removal effect of R-17F by performing GSL-glycan analysis before and after adding R-17F.

## 2. Results

### 2.1. Detection of Undifferentiated iPSCs with Human Chondrocytes by GSL-Glycome Analysis 

**Figure 1:**
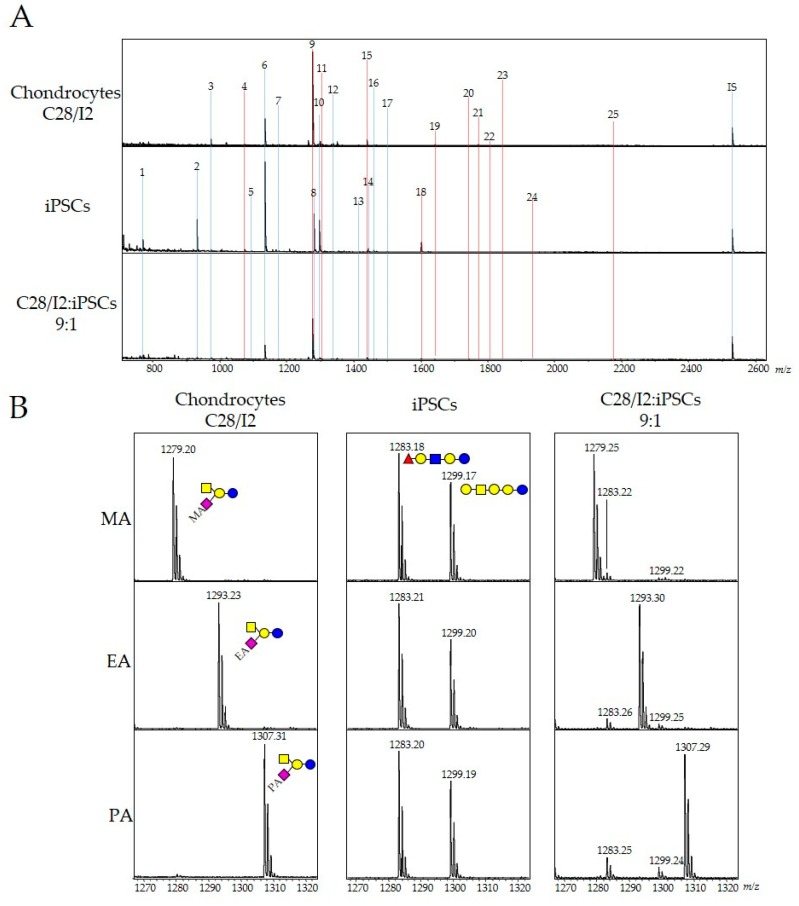
Analysis of GSL-glycans from iPSCs and chondrocytes using the aminolysis-SALSA method. (**A**) MALDI-TOF MS spectra of GSL-glycans in chondrocyte cells, iPSCs, and coexisting cells. (**B**) MALDI-TOF MS spectra of GSL-glycans from each cell type were acquired after derivatization with methylamine (MA), ethylamine (EA), or propylamine (PA) using the aminolysis-SALSA method, in the range of 1265−1325 *m*/*z*. The signal numbers correspond to those described in Table 1.

We previously analyzed the GSL-glycan profiles of two iPS cell lines (201B7 and 606A1) and confirmed that both GSL-glycan profiles were very similar (Figure S1). Based on this result, 606A1 was adopted for glycome analysis in this study. We investigated whether iPSC-specific GSL-glycans could be quantitatively detected when coexisting with chondrocytes by Matrix Assisted Laser Desorption/Ionization-Time of- Flight Mass (MALDI-TOF MS). Firstly, we analyzed GSL-glycans of iPSCs and chondrocytes (human chondrocyte cell line C28/I2). Cultured cells were homogenized, and the lipid fraction was recovered by ethanol precipitation. Intact glycans were released from GSLs by enzymatic digestion (cleavage by endoglycoceramidase I) as previously described [13]. GSL-glycans were purified by glycoblotting combined with aminolysis-SALSA using methylamine [14]. As shown in Figure 1, 18 signals corresponding to GSL-glycans were observed in samples from chondrocytes by MALDI-TOF MS analysis. The composition and quantitation of GSL-glycans in both chondrocytes and iPSCs (~5 × 10^4^ cells) were shown in Table 1. Gangliosides containing α2,3-linked sialic acid residues were highly expressed in chondrocytes and resulted in methylamide derivatives. One of the major gangliosides was GM2, which accounted for ~70% of total GSL-glycans. Meanwhile, 13 out of 17 GSL-glycans were neutral in iPSCs. Eight GSL-glycans were elevated 20-fold compared with levels in chondrocytes, and five GSL-glycans, of which four were neutral (Gb3, LNFP I, Gb5, and galactosyl LNFP I), and one ganglioside (SSEA-4) were measured at ≥0.1 pmol in 5 × 10^4^ cells. These results indicate that the expression levels of iPSC-specific GSL-glycans can be used to evaluate residual iPSCs with chondrocytes from MALDI-TOF MS spectra.

Next, we analyzed cellular GSL-glycans of chondrocytes with 10% iPSCs. As shown in Figure 1, most of the GSL-glycans identified from iPSCs or chondrocytes did not overlap under the coexisting conditions. Two glycan signals for methyl-amidated GM2 and LNFP I corresponding to chondrocytes and iPSCs were close in molecular weight, with only a 3 Da difference, making quantification difficult due to the small amount of LNFP I, which is expressed only in iPSCs under the coexisting conditions. Recently, we also reported that aminolysis-SALSA can be readily applied for the derivatization of α2,3-linked sialic acid residues using various amine reagents such as ethylamine and 2-aminoethanol [14]. Herein, we analyzed coexisting GSL-glycans by aminolysis-SALSA using ethylamine (EA) or propylamine (PA). MALDI-TOF MS spectra of GSL-glycans were shown in Figure 1B. LNFP I and Gb4 are neutral glycans, hence these signals were observed at m/z 1283.2 and 1299.2 (right panel) using all types of aminolysis. However, the signal for GM2 derived from chondrocytes was shifted upfield (EA = 14 Da, PA = 28 Da) from that of methyl-amidated GM2 (MA; left panel). The signals for ethyl- or propyl-amidated GM2 were completely separated from those of LNFP I and Gb4 in MALDI-TOF MS spectra. Based on these results, we decided to estimate residual iPSCs in chondrocytes using the ethylaminolysis-SALSA method.

### 2.2. Evaluation of Residual iPSCs among Human Chondrocytes by Cell Type-Specific GSL-Glycome Analysis

**Figure 2:**
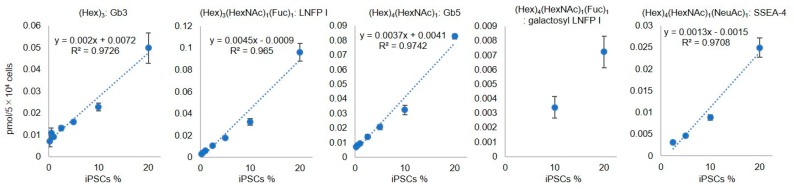
Linear dynamic ranges of the quantification of (Hex)_3_: Gb3, (Hex)_3_(HexNAc)_1_(Fuc)_1_: LNFP I, (Hex)_4_(HexNAc)_1_: Gb5, (Hex)_4_(HexNAc)_1_(Fuc)_1_: galactosyl LNFP I, and (Hex)_4_(HexNAc)_1_(NeuAc)_1_: SSEA-4 at various iPSC ratios (iPSC content = 20%, 10%, 5%, 2.5%, 1%, 0.5%, and 0.25%). Error bars indicate standard deviation (SD) for triplicate measurement.

Next, we examined whether iPSC-specific GSL-glycans could be quantitatively detected from chondrocytes containing iPSCs at various cell densities. GSL-glycans prepared from iPSCs and chondrocytes (approximately 5 × 10^4^ cells) were labeled with Nα-((aminooxy)acetyl) tryptophanylarginine methyl ester (aoWR) at the reducing end and α2,3-sialic acid was derivatized with ethylamine by ethylaminolysis-SALSA as described above. We prepared aoWR-labeled GSL-glycans mixed with iPSCs and chondrocytes at various ratios (iPSC content = 20%, 10%, 5%, 2.5%, 1%, 0.5%, 0.25% and 0.1%). A portion of the GSL-glycan mixture was spotted on a MALDI target plate containing 40 fmol of Neu5Ac_2_Gal_2_GlcNAc_2_ + Man_3_GlcNAc_1_ (A2GN1) as an internal standard. Quantitation of iPSC-specific GSL-glycans was performed by comparing the areas of the MS signals of each GSL-glycan with an internal standard. Spectra were obtained from ~1000 cells as shown in Figure S2. The iPS-specific glycans were well detectable up to 0.25%, but they were difficult to detect below that. We also acquired calibration curves using iPSC-specific glycans (Gb3, LNFP I, Gb5, galactosyl LNFP I, and SSEA-4) at different iPS cell densities. The three calibration curves for Gb3, LNFP I, and Gb5 displayed strong linear correlations (R^2^ > 0.97) between iPS cell density and quantitative glycan values, as shown in Figure 2, whereas the abundance of galactosyl LNFP I and SSEA-4 was insufficient to generate calibration curves. We also investigated the residual iPSCs in chondrocytes by flow cytometry. 1 × 10^6^ cells of iPS (606A1) were labeled with Cell Tracker Green CMFDA [7] and mixed with C28/I2 (1 × 10^6^ cells) at various ratios (iPSC content = 10%, 5%, 2.5%, 1%, 0.5%, and 0.1%). As shown in supplementary Figure S3, the residual iPSCs labeled with fluorescent probes were detectable in low concentration.

### 2.3. Evaluation of R-17F-Induced Cytotoxicity Toward iPSCs by GSL-Glycome Analysis

**Figure 3:**
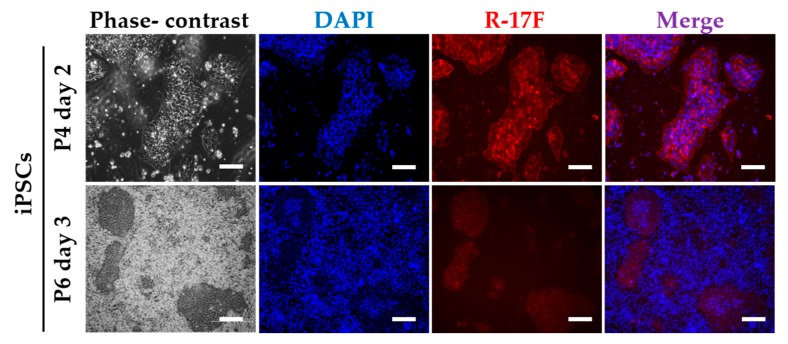
Evaluation of the ability of the R-17F antibody to bind iPSCs. Cultured iPS cell lines 606A1 (P4, day2) and 201B7 (P6, day3) were incubated with R-17F (10 µg/mL), followed by stained with 4’,6-diamidino-2-phenylindole (DAPI, Blue) and incubation with secondary antibody (Alexa Fluor 594-conjugated goat anti-mouse IgG antibody, Red). Scale bar = 500 μm.

**Figure 4:**
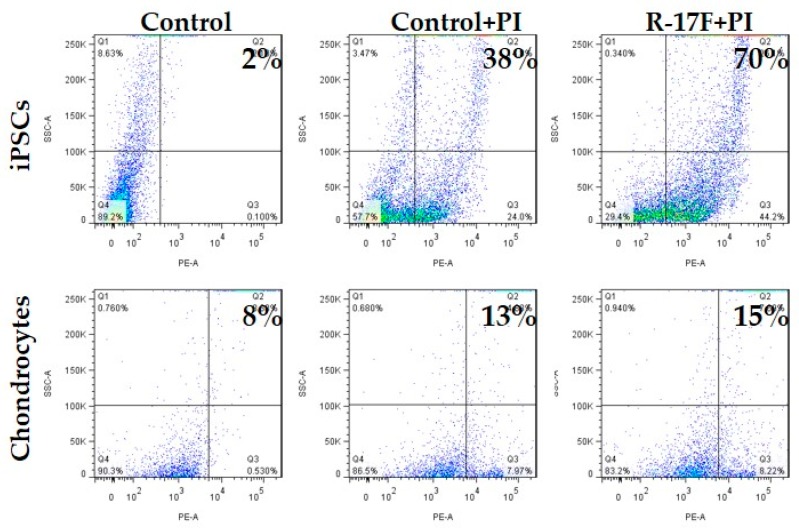
Evaluation of the cytotoxicity of R-17F against iPSCs and chondrocytes. Both iPSC (606A1) and chondrocyte (C28I2) cell lines were treated with R-17F, stained with PI, and analyzed by flow cytometry.

R-17F is an antibody that detects undifferentiated iPSCs by recognizing the LNFP I of GSL, and consequently exerts cytotoxic activity [9]. Therefore, we tried to investigate the removal of residual iPSCs via R-17F-induced cytotoxicity toward co-cultured cells for iPSCs and chondrocytes. Firstly, the binding ability of R-17F to iPSCs was evaluated using immunohistological staining. Cultured iPSCs (606A1, P4, and 207B7, P6) were fixed in 4% paraformaldehyde (PFA) and incubated with R-17F (10 µg/mL) at 4 °C overnight, then incubated with the secondary antibody (Alexa Fluor 594-conjugated goat anti-mouse IgG antibody). The labeling cells were imaged by a fluorescence microscope (BZ-X710, KEYENCE, Osaka, Japan). Figure 3 shows that R-17F bound to all iPSC colonies, as evidenced by the fluorescence signal. In the 201B7 cells, differentiated iPSCs surrounded undifferentiated iPSC colonies, but only undifferentiated iPSC colonies were R-17F-positive. Next, we confirmed the cytotoxicity of R-17F toward either iPSCs or chondrocytes. After incubation of either iPSCs or chondrocytes (C28/I2) with R-17F at a concentration of 100 μg/mL for 45 min, propidium iodide (PI)-positive (dead) cells were quantified by flow cytometry. The cytotoxic activity of R-17F against iPSCs was estimated to be >70%, compared with only 38% for control iPSCs, as shown in Figure 4. In sharp contrast with iPSCs, PI-positive cells showed almost no differences between R-17F-induced chondrocytes (15%) and controls (13%). R-17F exerted a specific cytotoxic effect against iPSCs, but not chondrocytes. We also evaluated the cytotoxicity of R-17F against iPSC colonies. After incubation with 500 μg/mL of R-17F for 24 h, necrosis of colonies was confirmed by microscopy (Figure S4).

Next, we prepared co-cultured chondrocytes containing iPSCs at various ratios and analyzed GSL-glycans to evaluate R-17F-induced cytotoxicity against iPSCs. Chondrocytes (C28/I2) and iPSCs (606A1) were cultured with each other for 4–5 days until 80% confluence. In addition, we also compared the GSL-glycan of either C28/I2 or iPSCs at three different experiments and confirmed that GSL-glycan profiles were cellular specific and similar regardless differences in cell passage (Figure S5). Cells were detached and the number of cells was determined, then mixed at various ratios (iPSC rate = 100%, 20%, 10%, 2.5%, 1%, 0.5%, and 0%), centrifuged at 200 g for 3 min, combined with essential 8 medium containing 50 U/mL penicillin and 50 mg/mL streptomycin and seeded vitronectin coated 24 well plate (seeding density, 7.5 × 10^4^ cells/well). The medium was changed daily and cultured for 4 days. Co-cultured cells were collected and the ~5 × 10^4^ cells were used for GSL-glycome analysis by the glycoblotting-SALSA method. As shown in Figure S6A, favorable GSL-glycan profiles were obtained using 5 × 10^4^ cells, and iPSC-specific LNFP I could be detected using 1 × 10^4^ or more cells. The limit of detection was also examined using aoWR-labeled GSL-glycan derived from 5 × 10^4^ iPSCs. Most GSL-glycans could be detected even after 500-fold dilution, equating to ~100 cells, with 4 fmol of A2GN1 as an internal standard (Figure S6B). MALDI-TOF MS spectra of GSL-glycome samples derived from co-cultured cells at various ratios were shown in Figure S7. As in the case of co-cultured cells, the signals for iPSC-specific GSL-glycans decreased gradually depending on the iPSC ratio. Gb3 at m/z 934, LNFP I at m/z 1283, and SSEA-4 at m/z 1617 were detectable from co-cultured cells containing 1% of iPSCs.

**Figure 5:**
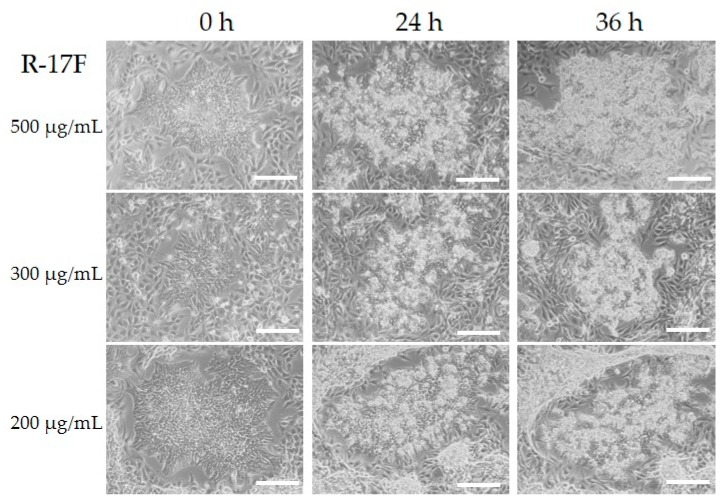
Images of iPSC colonies and chondrocytes (C28/I2) after treatment with R-17F at various concentrations. iPSCs and chondrocytes (C28/I2) were cultured for 3 days, followed by adding R-17F at the concentration of 200–500 μg/mL. All cells were monitored under a microscope. Scale bar = 500 μm.

**Figure 6:**
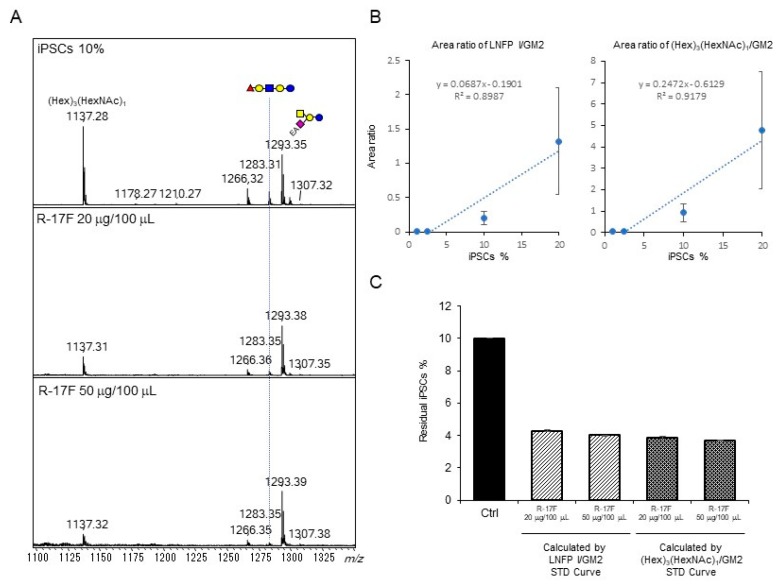
MALDI-TOF MS analysis of GSL-glycans from co-cultured cells (chondrocytes and iPSCs) treated with R17-F antibody. (**A**) MALDI-TOF MS spectra of co-cultured cells and antibody-treated cells. (**B**) Linear dynamic ranges of LNFP I/GM2 and (Hex)_3_(HexNAc)_1_/GM2 area ratios at different iPSC co-cultured conditions (iPSC content = 20%, 10%, 2.5%, and 1%). (**C**) Evaluation of residual iPSCs co-cultured with chondrocytes. The residual iPSC percentage was calculated using calibration curves. Error bars indicate standard deviation (SD) for triplicate measurement.

Next, we attempted to evaluate R-17F-induced cytotoxicity toward residual iPSCs by GSL-glycome analysis. Both iPSCs and chondrocytes (C28/I2) were seeded on a 24-well plate (iPSC seeding rate = 10%) and cultured for 3 days, followed by addition of R-17F at a concentration of 200−500 μg/mL. Microscopy examination confirmed that iPSC colonies withered gradually over time, and almost all colonies were necrotic after 36 h after addition of R-17F antibody, whereas most chondrocytes remained intact at this timepoint (Figure 5). The decrease in iPSC-specific GSL-glycans following R-17F treatment was shown in Figure 6A. The signal intensity of LNFP I at *m*/*z* 1283, which is a glycoepitope of the R-17F antibody, was decreased; as was that of (Hex)_3_(HexNAc)_1_ at *m*/*z* 1137. By contrast, the signal for ethyl-amidated GM2 (*m*/*z* 1293) derived from chondrocytes was not affected by the cytotoxicity of the R-17F antibody.

**Figure 7:**
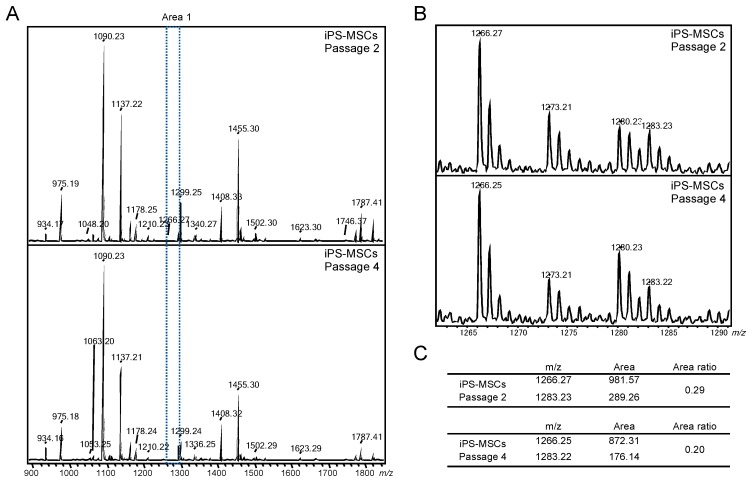
(**A**) MALDI-TOF MS spectra of GSL-glycans in passage 2 and 4 iPS-MSCs. (**B**) Close-up views in Area 1. (**C**) The area ratio of *m*/*z* 1283 and *m*/*z* 1266 in passage 2 and 4 iPS-MSCs.

We then attempted to estimate residual iPSCs after addition of R-17F by GSL-glycan analysis. To this end, we prepared calibration curves using chondrocytes co-cultured with iPSCs at various ratios. Calibration curves were created using the ratio of the signal for iPSC-specific LNFP I or (Hex)_3_(HexNAc)_1_ and ethyl-amidated GM2 derived from chondrocytes. In the presence of R-17F antibody at concentrations of 200 or 500 μg/mL, the relative amount of residual iPSCs was decreased similarly from 10% to ~4% (Figure 6C). Next, we evaluated the change iPSC-specific glycans during the differentiation from iPSCs to mesenchymal stem cells-like cells (iPSC-MSCs). iPSCs were maintained and passaged 3−5 times under feeder-free conditions in which vitronectin coating and Essential 8 medium were used according to a protocol described previously [15]. We then subcultured undifferentiated iPSCs and induced their transformation into iPSC-MSCs until passage 6 using a previously described method [16]. These mesenchymal stem cells-like cells (P2, P4, and P6) expressed few amounts of Nanog, Oct 3/4, and Sox 2, which were frequently used as an undifferentiated marker, and there was no significant difference in gene expression between P2, P4, and P6. (Figure S8). iPS-MSCs P6 were also analyzed by flow cytometry using MSC markers, confirmed that they had MSC-like properties (Figure S9). Second, GSL-glycan analysis of iPSC-MSCs at different passage times (P2 and P4) was performed in the same manner as described above. The results revealed that the expression of iPS-specific LNFP I were gradually decreased during repeating passages of iPSC-MSCs (Figure 7). These results suggested that the residual iPSCs may be decreased depending on the passage times.

## 3. Discussion

Since Gropp et al. reported that a teratoma can be established by only a few hundred iPSCs, it is a prerequisite that final products used in regenerative medicine do not contain residual iPSCs when using iPSC-derived cells [17]. To overcome this problem, several strategies have been reported that promote the selective removal of residual iPSCs from a population of differentiated cells, such as the introduction of suicide genes into iPSCs [18], alteration of cell culture conditions [6], and cell sorting using antibodies against cell surface antigens [8]. In these reports, detection procedures for undifferentiated cells included fluorescent labeling, flow cytometry, and methods for confirming teratoma formation in vivo. In previous reports, undifferentiated cells were detected in retinal pigment epithelial cells by flow cytometry using anti-TRA-1-60 antibody [19], and the detection sensitivity was 0.1%. Although flow cytometry is a highly sensitive and useful method, it is an indirect method for evaluating cells labeled with antibodies against a cell surface marker, hence there is the possibility of false negatives. In addition, analysis of the results may be greatly influenced by gating. Although animal experiments for confirming teratoma formation are generally highly reliable, they are costly and time-consuming. 

A flow cytometry method has been used for detecting undifferentiated iPSCs. However, flow cytometry requires ~5 × 10^5^ cells and cell-specific labeling according to each cell type [20]. Using our method that focuses directly on GSL-glycans on the cell surface, we could detect undifferentiated iPSC-specific glycans prepared from only 5 × 10^4^ cells. According to a previous report, teratomas may be formed from as few as 100 iPSCs [17]. In the present study, most GSL-glycans from 100 iPSCs could also be detected by MALDI-TOF MS. Moreover, iPSC-specific glycans were detected even by chondrocytes co-cultured with iPSCs. 

The advantage of glycan analysis is not only that it is possible using very few cells, but also that multiple iPSC-specific GSL-glycans can be simultaneously detected. In chondrocytes co-cultured with iPSCs, it is very important to discriminate cell type-specific glycans using the aminolysis-SALSA method. Using various amine analogues such as MA, EA, and PA can also avoid overlapping peaks according to desirable GSL-glycans. However, regarding sensitivity, flow cytometry could detect iPSCs present at only 0.1%, whereas the limit for glycome analysis was 0.25%, hence flow cytometry was superior in terms of sensitivity. Even so, glycoblotting-SALSA technology can facilitate large-scale GSL-glycan analysis for evaluation of residual iPSCs. However, detection of undifferentiated iPSCs in co-existing cells by flow cytometry requires complicated pretreatment process: 1) cells dissociation by enzymatic digestion, 2) fixation of a single cell, 3) iPSC-specific labeling using primary antibody such as TRA-1-60, SSEA-5, 4) immunofluorescent labeling using secondary antibody. Furthermore, there is a problem that cartilaginous particles generated from iPSCs dissociate into a single cell. On the other hand, GSL-glycome analysis did not require complicated process as described above. Undifferentiated iPSC-specific GSL-glycans could be observed from co-cultured cells or cartilaginous particles. Moreover, glycoblotting-SALSA technology could allow for the performance of large scale GSL-glycomics for evaluation of the residual iPSCs.

We were also able to evaluate the removal of iPSCs due to the cytotoxicity of the R-17F antibody. Our results showed that levels of LNFP I decreased from 10% to 4% due to cytotoxicity of the R-17F antibody. Moreover, we confirmed that LNFP I could be detected from iPS-MSCs with very low expression of undifferentiated markers such as Nanog, Oct 3/4 and Sox 2. We believe that general undifferentiated marker genes combined with GSL-glycan analysis is useful for evaluating residual iPSCs.

Since R-17F is a cellular cytotoxic antibody that recognizes the iPS-specific LNFP I, evaluation of necrosis of undifferentiated iPSCs by GSL-glycome analysis is entirely feasible. Almost all iPSCs appeared to be necrotic according to microscopy observations, but our GSL-glycome analysis indicated that a small number of iPSCs remained. A previous study reported that it took 3 days for necrosis of iPS colonies due to cytotoxicity of the R-17F antibody, and the use of a secondary antibody recognizing R-17F enhanced the cytotoxicity [9]. Recently, Ben-David et al. reported iPSCs-specific inhibitors “PluriSIns”, which does not affect differentiated iPSCs [21]. Small molecules (PluriSIns) may also be effective to prevent teratoma formation. This multiple and large scale GSL-glycomic method may be able to use for optimization of cytotoxicity under various reaction conditions. Cellular GSL-glycome analysis combined with the aminolysis-SALSA method may provide a powerful tool with which to confirm the removal of iPSCs from differentiated cells, and thereby ensure safer iPSC-based regenerative medicine.

## 4. Materials and Methods 

### 4.1. Experimental Animals and Materials

Human-induced pluripotent stem (iPS) cell lines (201B7 and 606A1) were purchased from Riken (Ibaraki, Japan). The human chondrocyte cell line (C28/I2) was purchased from Merck (Darmstadt, Germany). R-17F was provided by Dr. Kawasaki, Ritsumeikan University (Shiga, Japan). 4’,6-diamidino-2-phenylindole (DAPI) and Propidium iodide (PI) was purchased from DOJINDO (Kumamoto, Japan). Alexa Fluor 594-conjugated goat anti-mouse IgG antibody was purchased from Invitrogen (Tokyo, Japan). BlotGlyco beads were from Sumitomo Bakelite Company Ltd. (Tokyo, Japan). Other solvents and reagents were of the highest grade commercially available.

### 4.2. Cell Culture

The 201B7 and 606A1 cells were cultured in a feeder-free medium that included Essential 8 (Invitrogen) containing 50 U/mL penicillin and 50 mg/mL streptomycin on cell culture plates coated with vitronectin (Invitrogen). Undifferentiated colonies were passaged every 3–4 days using Versene solution (Thermo Fisher Scientific, Tokyo, Japan). C28/I2 cells were cultured in Dulbecco’s Modified Eagle Medium (DMEM, Wako, Osaka, Japan) supplemented with 10% fetal bovine serum (FBS), 50 U/mL penicillin, and 50 mg/mL streptomycin. C28/I2 cells were passaged every 4 days using trypsin- ethylenediaminetetraacetic acid (EDTA) solution. 

### 4.3. GSL-Glycome Analysis of iPSCs Co-Cultured with Chondrocytes

iPSCs (606A1) and chondrocytes (C28/I2) were cultured with each other until 80% confluent. Cultured cells were washed three times with phosphate-buffered saline (PBS), detached with a cell scraper, and collected in a centrifuge tube. Viable cells were stained with Trypan Blue (Invitrogen), and the number of cells was counted using a Countess Automated Cell Counter (Invitrogen). Next, iPSCs and chondrocytes were mixed at different ratios (iPSC content = 100%, 50%, 30%, 20%, 10%, 5%, 2.5%, and 0%; total cells = 1 × 10^6^). Mixed cells were centrifuged at 440 *g* for 5 min and the supernatant was aspirated. After rinsing with 1000 μL PBS and centrifuging at 800 g for 5 min, 100 μL cultured cells in PBS were homogenized using a BIORUPTOR II instrument (CosmoBio, Tokyo, Japan) at 4 °C, followed by addition of a 4-fold volume of ethanol in the cell lysate. After ethanol precipitation, the resulting mixtures were centrifuged at 14,000 *g* for 20 min. Supernatants containing glycosphingolipids (GSLs) were recovered and concentrated with a centrifugal evaporator. The lipid fraction was resuspended in 50 mM acetate buffer, pH 5.5 (48 μL), containing 0.2% Triton X-100 (Sigma-Aldrich, Tokyo, Japan) as a surfactant, followed by the addition of 2 μL endoglycosylceramidase (EGCase) I (25 mU) to release intact glycans from GSLs, as previously described [13,22]. Prepared GSL-glycans were subjected to glycoblotting aminolysis-SALSA methods [14]. Briefly, intact GSL-glycans containing internal standards Neu5Ac_2_Gal_2_GlcNAc_2_ + Man_3_GlcNAc_1_ (A2GN1) were captured on 5 mg BlotGlyco beads (Sumitomo Bakelite Company Ltd., Tokyo, Japan). Unreacted hydrazide groups on beads were capped by acetylation with 10% acetic anhydride in methanol. Next, 100 μL of the first alkylamidation solution was added to wells containing beads and incubated at room temperature for 1 h with shaking in a microplate mixer (TOMY, Tokyo, Japan). Excess amidation reagents were removed, and beads were washed with 200 μL MeOH. For the aminolysis-SALSA procedure, beads were washed three times with 200 μL of 2.9 M amine reagent (methylamine, ethylamine, or propylamine), MeOH, and water. Finally, capturing GSL-glycans were released from BlotGlyco beads and derivatized with aoWR reagents by an imine-exchange reaction. The recovered solution was applied to a hydrophilic interaction liquid chromatography (Waters, Milford, MA, USA) plate to remove the excess aoWR reagents. The solution containing GSL-glycans was mixed with 2,5-dihydrobenzoic acid solution (10 mg/mL in 30% acetonitrile aq) and subjected to matrix-assisted-laser-desorption/ionization–time-of-flight mass (MALDI-TOF MS) analyses as previously described [12]. Briefly, all measurements were performed on an Ultraflex II TOF/TOF-MS instrument equipped with a reflector and controlled by FlexControl 3.0 software (Bruker Daltonics, Bremen, Germany). All MS spectra were obtained in reflectron mode with an acceleration voltage of 25 kV, a reflector voltage of 26.3 kV, and a pulsed-ion extraction of 160 ns in positive ion mode. Masses were annotated using FlexAnalysis 3.0 software (Bruker Daltonics, Bremen, Germany) and SphinGOMAP (http://www.sphingomap.org/ accessed on 31 October 2019) online databases for structural identification of GSL-glycans.

### 4.4. Flow Cytometry Analysisof the Cytotoxic Activity of R-17F

Cytotoxicity against iPSCs was subsequently evaluated. Specifically, iPSCs (606A1, 1 × 10^5^ cells) were incubated with R-17F (100 µg/mL) in PBS containing 2% FBS and 25 mM 4-(2-Hydroxyethyl)piperazine)-1-ethanesulfonic acid (HEPES, SIGMA-ALDRICH, St. Louis, USA) at 4 °C for 45 min. After washing by centrifugation, pellets were resuspended in PBS containing 4 µmol/L PI. After incubating at 37 °C for 15 min and washing by centrifugation, stained cells were analyzed using a BD FACSCANTO II instrument (BD Biosciences, NJ, USA). The effect of R-17F on human chondrocytes was evaluated by flow cytometry in the same manner as for iPSCs using the C28I2 chondrocyte cell line. Data retrieved from cell sorting was analyzed using Flowjo software (Tree Star, Ashland, USA).

### 4.5. GSL-Glycome Analysis of the Cytotoxic Activity of R-17F in Chondrocytes Co-Cultured with iPSCs

For GSL-glycome analysis, iPSCs (606A1) and chondrocytes (C28/I2) were co-cultured in a 24-well plate (number of seeded cells = 0.75 × 10^4^ and 0.75 × 10^5^). After culturing for 3 days, R-17F was added at a concentration of 200−500 μg/mL iPS medium. One day later, cells were washed with PBS, and all cells were detached with a cell scraper and collected in a microtube (total cells = 5 × 10^5^). The supernatant containing GSL-glycans was separated by ethanol precipitation, and released GSL-glycans were subjected to glycoblotting as described above. 

### 4.6. In Vitro Differentiation of iPSCs into iPSC-Derived MSCs

Undifferentiated iPSC colonies cultured in a feeder-free medium were treated with Versene solution for 5 min at 37 °C, dissociated by gentle pipetting with Essential 8, and then cells were seeded onto gelatin-coated plates at 1 × 10^4^ cells/cm². Three days later, the medium was changed to MSC induction medium consisting of DMEM-HG (Gibco), 10% FBS, 1% nonessential amino acids, 1% penicillin-streptomycin, and 5 ng/mL human recombinant basic fibroblast growth factor (bFGF), as previously described [16]. Cells were propagated to 80% confluency in a humidified atmosphere at 37 °C and 5% CO_2_. With subsequent passaging (p1–p2) onto non-coated tissue culture plates using 0.25% trypsin, iPSC-mesenchymal stem cells-like cells (MSCs)-like populations acquired a homogenous, fibroblast-like morphology. For routine expansion, cells were plated at 1 × 10^4^ cells/cm² and maintained in MSC induction medium.

### 4.7. RNA Extraction and Quantitative Real-Time Polymerase Chain Reaction

Total RNA was extracted from iPSCs, iPS-MSC, and hBMSCs using TRIzol reagent (Life Technologies, Carlsbad, CA, USA) and purified with column cleanup (Qiagen, Hilden, Germany). To synthesize cDNA, reverse transcription was performed using the QuantiTect Reverse Transcription Kit (Qiagen) according to the manufacturer instructions. Quantitative real-time polymerase chain reaction (PCR) was performed using the SYBR Green I-based RT-PCR Master Mix and the Thermal Cycler Dice Real-Time System II (model TP900; Takara Bio, Shiga, Japan). The primers used for quantitative real-time PCR are listed in Supplemental Table S1. The gene expression levels were normalized to GAPDH. 

## Figures and Tables

**Table 1:** The glycan composition and quantitation of GSL-glycans in both chondrocytes and iPSCs.

No.	Glycan Composition	Class	m/z	Chondrocytes (pmol/5 × 10^4^)			iPSCs (pmol/5 × 10^4^)			Relative Amount iPSCs
1	(Hex)_2_	Neutral	772.39	0.06	±	0.01	0.22	±	0.02	3.6
2	(Hex)_3_	Neutral	934.47	0.04	±	0.01	0.80	±	0.08	21.2
3	(Hex)_2_(HexNAc)_1_	Neutral	975.5	0.19	±	0.04	0.06	±	0.00	0.3
5	(Hex)_4_	Neutral	1096.53	0.00	±	0.00	0.02	±	0.01	INF
6	(Hex)_3_(HexNAc)_1_	Neutral	1137.56	0.99	±	0.18	2.93	±	0.32	3.0
7	(Hex)_2_(HexNAc)_2_	Neutral	1178.59	0.04	±	0.01	0.04	±	0.00	1.1
8	(Hex)_3_(HexNAc)_1_(Fuc)_1_	Neutral	1283.63	0.00	±	0.00	1.36	±	0.18	INF
10	(Hex)_4_(HexNAc)_1_	Neutral	1299.67	0.01	±	0.01	1.15	±	0.13	174.5
12	(Hex)_3_(HexNAc)_2_	Neutral	1340.67	0.09	±	0.02	0.03	±	0.00	0.3
13	(Hex)_3_(HexNAc)_1_(Fuc)_2_	Neutral	1429.7	0.00	±	0.00	0.01	±	0.01	INF
14	(Hex)_4_(HexNAc)_1_(Fuc)_1_	Neutral	1445.71	0.00	±	0.00	0.14	±	0.01	INF
16	(Hex)_5_(HexNAc)_1_	Neutral	1461.71	0.01	±	0.01	0.07	±	0.01	10.5
17	(Hex)_4_(HexNAc)_2_	Neutral	1502.72	0.01	±	0.01	0.02	±	0.01	1.5
4	(Hex)_2_(α2,3NeuAc)_1_	Ganglioside	1076.57	0.04	±	0.01	0.07	±	0.01	1.7
9	(Hex)_2_(HexNAc)_1_(α2,3NeuAc)_1_	Ganglioside	1279.67	4.25	±	0.87	0.00	±	0.00	0
11	(Hex)_2_(HexNAc)_1_(α2,6NeuAc)_1_	Ganglioside	1307.7	0.07	±	0.01	0.00	±	0.00	0
15	(Hex)_3_(HexNAc)_1_(α2,3NeuAc)_1_	Ganglioside	1441.73	0.27	±	0.05	0.06	±	0.01	0.2
18	(Hex)_4_(HexNAc)_1_(α2,3NeuAc)_1_	Ganglioside	1603.78	0.00	±	0.00	0.46	±	0.05	INF
19	(Hex)_3_(HexNAc)_2_(α2,3NeuAc)_1_	Ganglioside	1644.81	0.05	±	0.01	0.00	±	0.00	0
20	(Hex)_3_(HexNAc)_1_(α2,3NeuAc)_2_	Ganglioside	1745.86	0.01	±	0.01	0.00	±	0.00	0
21	(Hex)_3_(HexNAc)_1_(α2,3NeuAc)_1_(α2,6NeuAc)_1_	Ganglioside	1773.88	0.06	±	0.01	0.00	±	0.00	0
22	(Hex)_4_(HexNAc)_2_(α2,3NeuAc)_1_	Ganglioside	1806.85	0.02	±	0.00	0.00	±	0.00	0
23	(Hex)_3_(HexNAc)_3_(α2,3NeuAc)_1_	Ganglioside	1847.87	0.01	±	0.01	0.00	±	0.00	0
24	(Hex)_4_(HexNAc)_1_(α2,3NeuAc)_1_(α2,6NeuAc)_1_	Ganglioside	1935.87	0.00	±	0.00	0.03	±	0.00	INF
25	(Hex)_5_(HexNAc)_3_(α2,3NeuAc)_1_	Ganglioside	2172	0.02	±	0.01	0.00	±	0.00	0
	Total	-	-	6.25	±	1.20	7.45	±	0.83	1.2

## Supplementary Materials

Supplementary materials can be found at https://www.mdpi.com/1422-0067/21/1/231/s1.
