# Supplementary material for: Evaluation of Residual Human-Induced Pluripotent Stem Cells in Human Chondrocytes by Cell Type-Specific Glycosphingolipid Glycome Analysis Based on the Aminolysis-SALSA Technique
Source: Int J Mol Sci. 2019 Dec 28;21(1):231. doi: 10.3390/ijms21010231 (PMC6982033; doi:10.3390/ijms21010231)
Supplement: Supplementary file 1 [file ijms-21-00231-s001.pdf]

Supplementary Figure S1

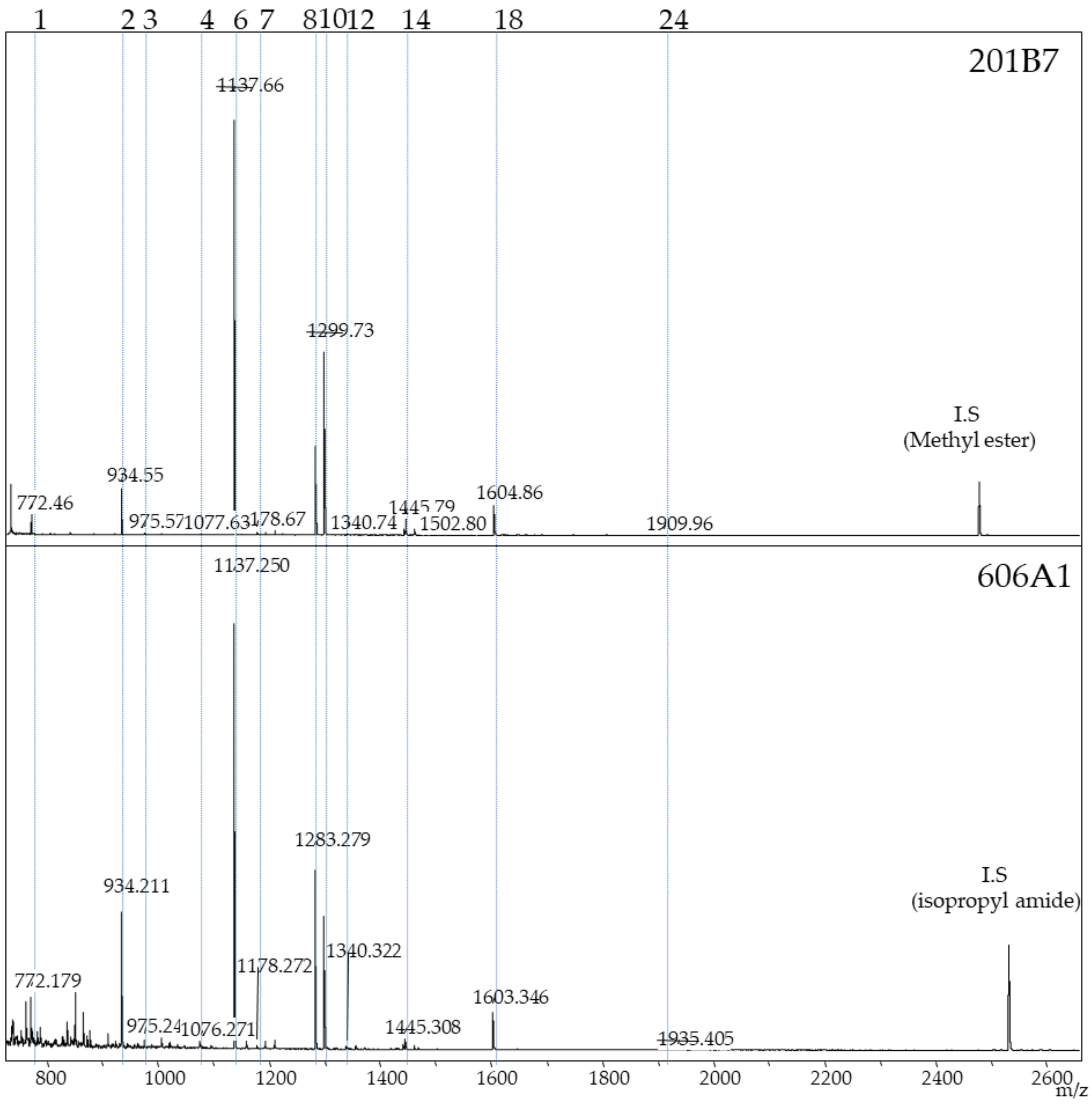

Figure S1. MALDI-TOF MS spectra of GSL-glycans in iPS cell lines 201B7 and 606A1. The signal numbers correspond to those described in Table 1.

Supplementary Figure S2

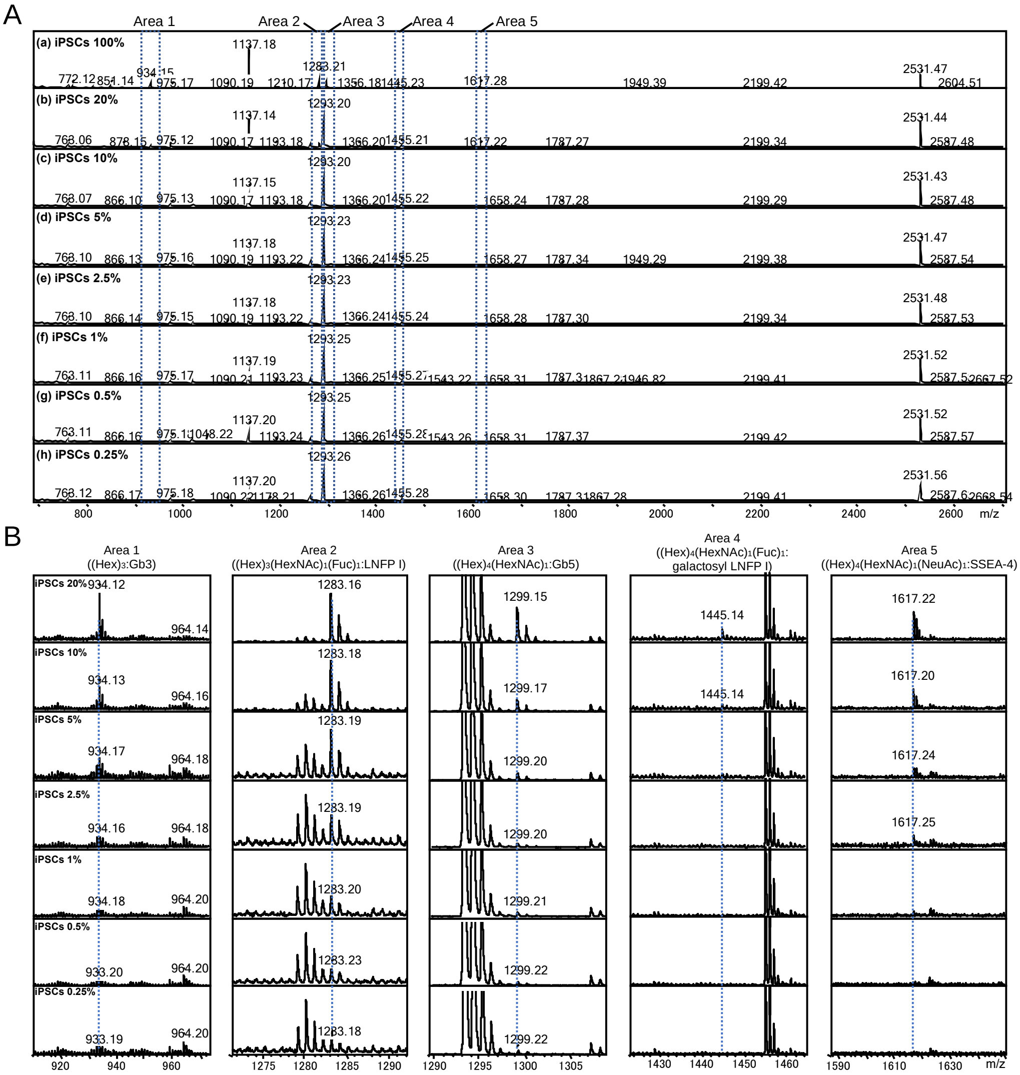

Figure S2. MALDI-TOF MS spectra showing GSL-glycans in C28/I2 containing iPSCs at various cell densities. (A) MALDI-TOF MS spectra of GSL-glycans in various iPSCs containing conditions (iPSCs 20%, 10%, 5%, 2.5%, 1%, 0.5%, and 0.25%). (B) Close-up views in areas 1-5.

Supplementary Figure S3

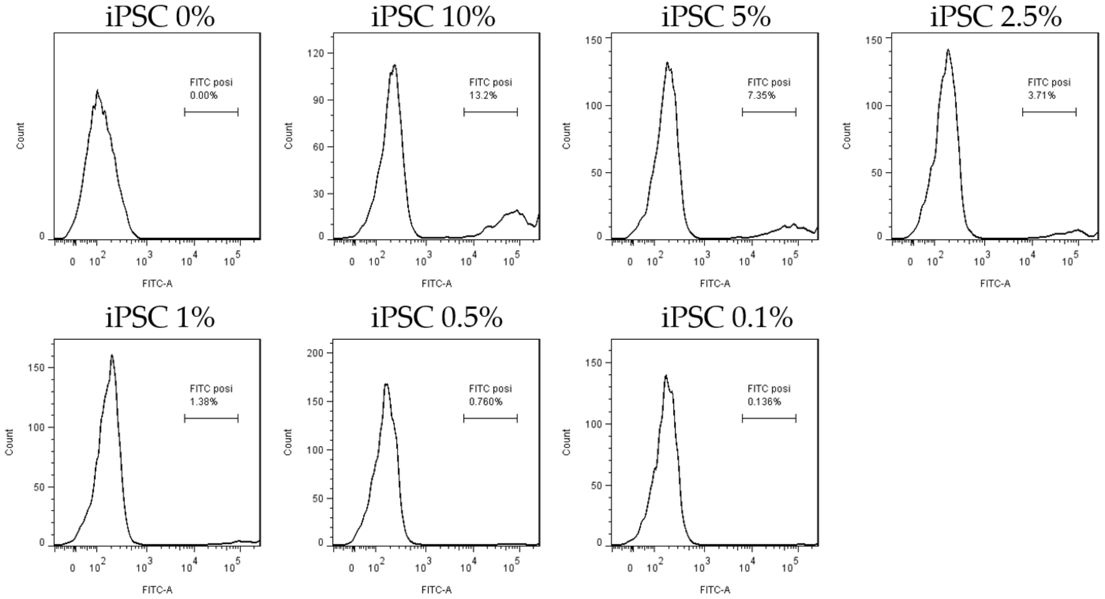

Figure S3. The residual iPSCs in chondrocytes by flow cytometry. iPSCs (606A1) labeled with Cell Tracker Green CMFDA were mixed in a considerably small proportion (from 10-0.1%) with chondrocytes, and $1x10^6$ cells were analyzed by flow cytometry.

Supplementary Figure S4

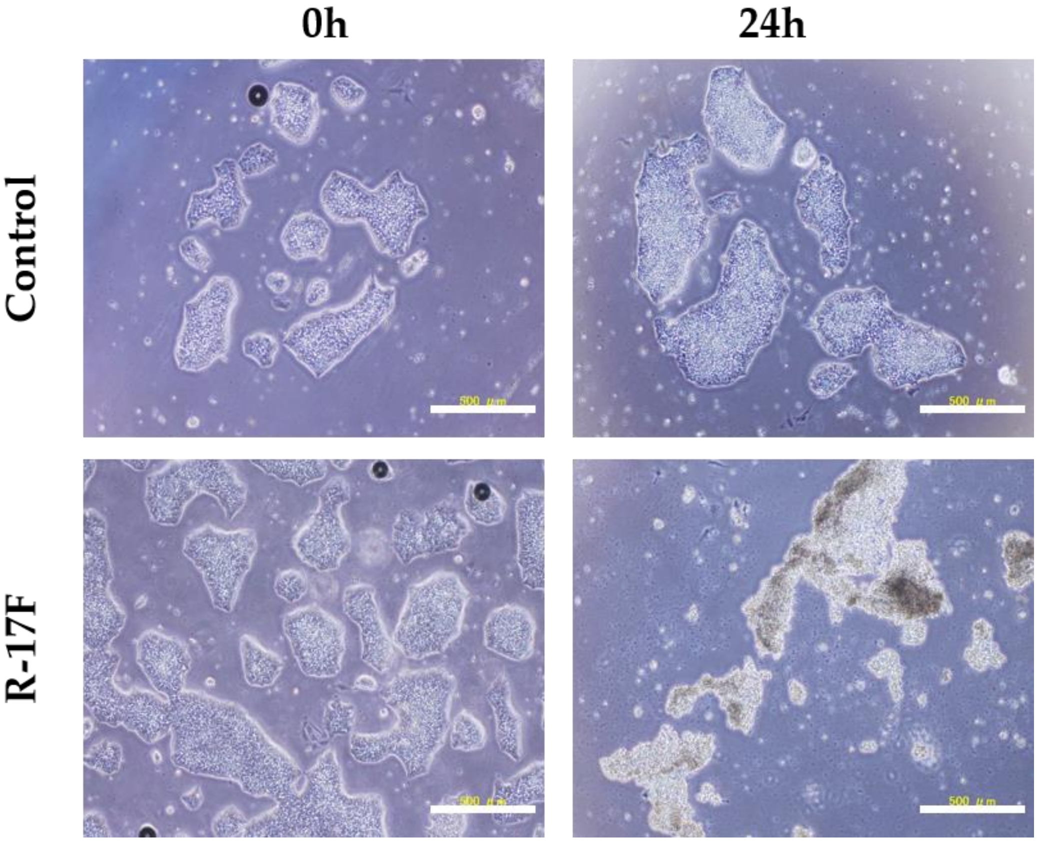

Figure S4. The evaluation of cytotoxicity of R-17F against iPSC colonies. iPSC colonies in the presence of R-17F were monitored under a microscope at every 12h.
Scale bar = 500 μm

Supplementary Figure S5

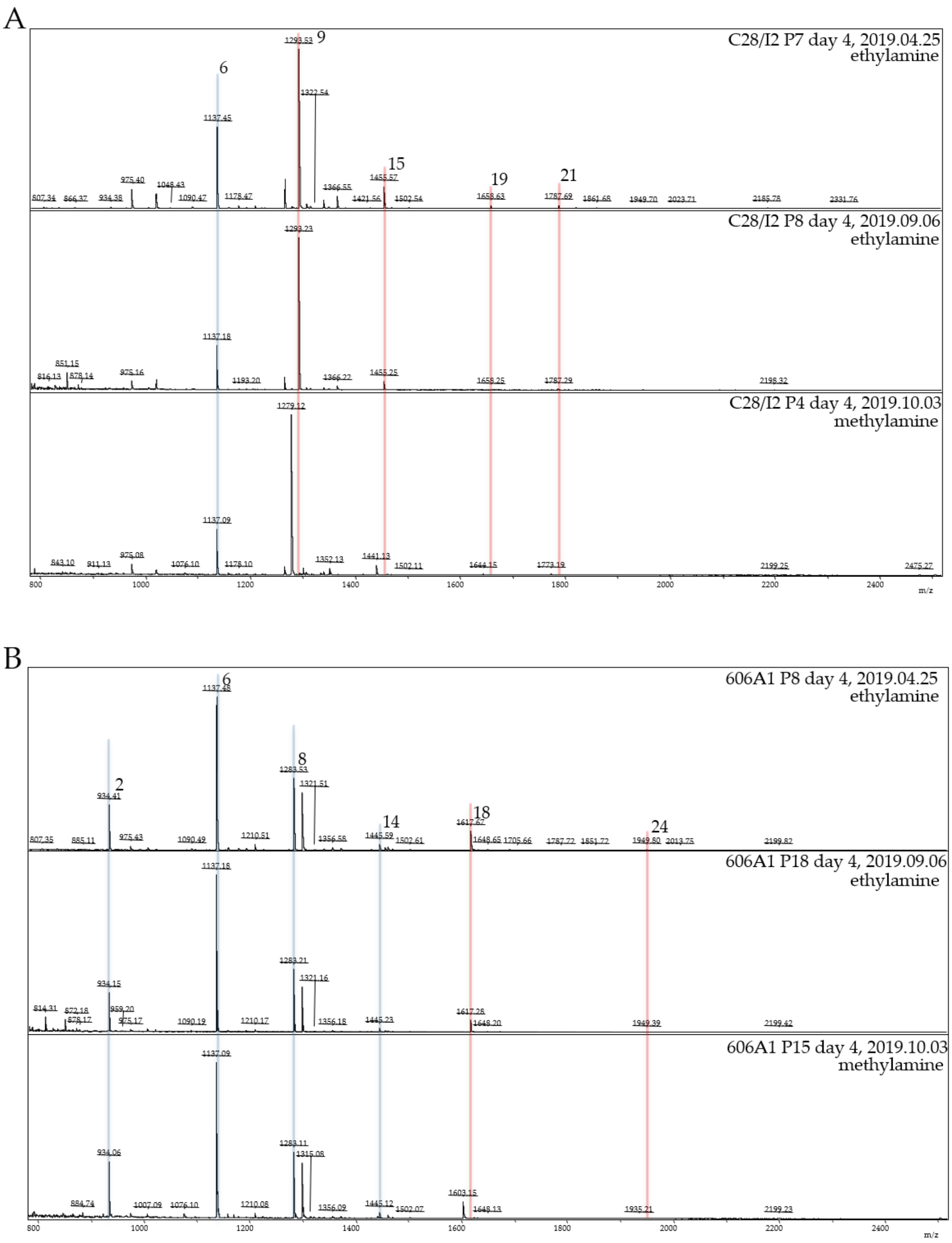

Figure S5. **MALDI-TOF MS spectra of GSL-glycans from chondrocyte cell lines and iPSCs.** GSL-glycans from chondrocyte cell lines (C28/I2) and iPSCs (606A1) were prepared by aminolysis-SALSA method with either ethylamine or methylamine. The signal numbers correspond to those described in Table 1.

Supplementary Figure S6

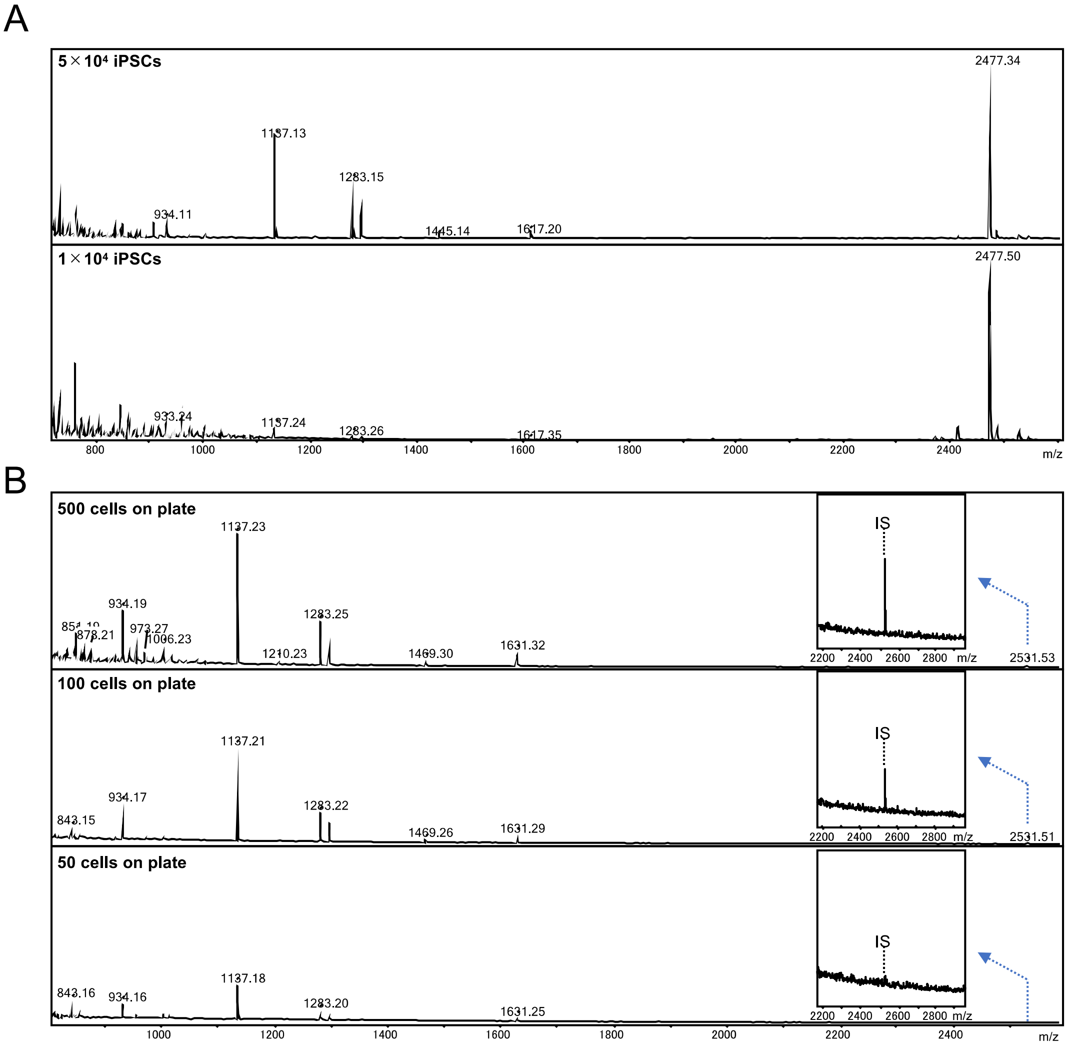

Figure S6. Examination of the detection limit of MALDI-TOF MS analysis. (A) MALDI-TOF MS spectra of GSL-glycans of 5×10^4 or 1×10^4 iPSCs prepared by solid-phase aminolysis-SALSA. The signal of methyl-esterified A2GN1 as an external standard is observed at *m/z* 2477. (B) MALDI-TOF MS spectra of 500 cells of iPS and of diluted samples (100 and 50 cells) on MALDI target plate.

Supplementary Figure S7

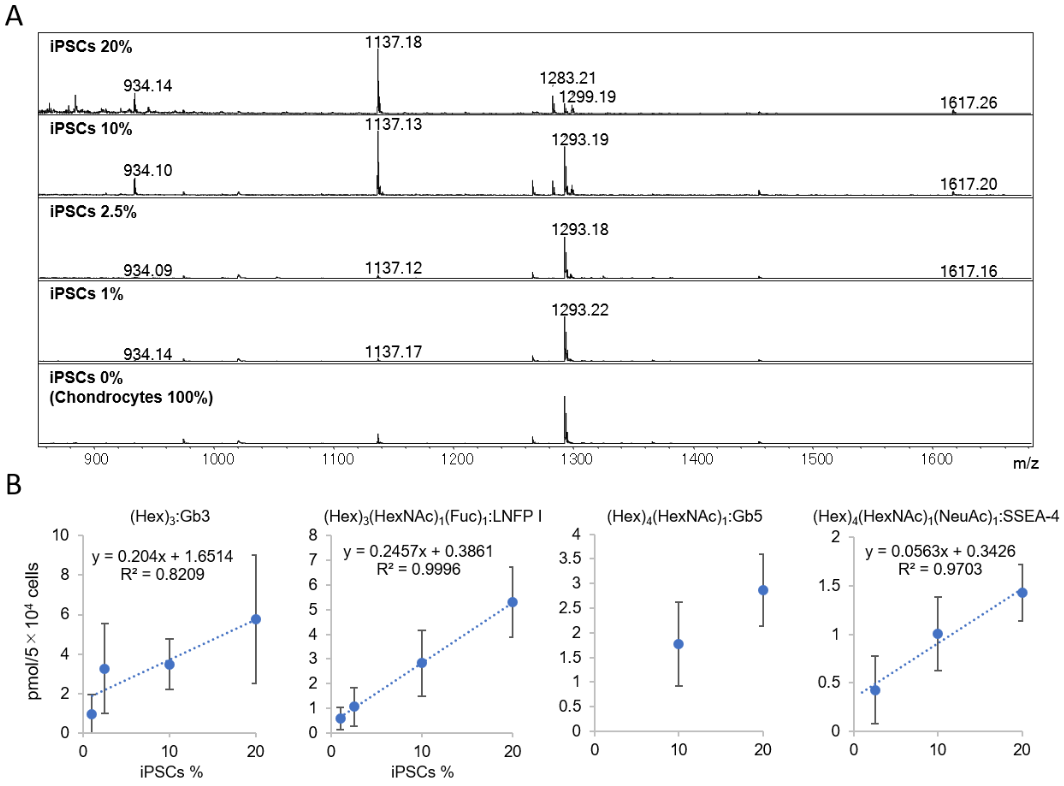

Figure S7. MALDI-TOF MS spectra of GSL-glycans in co-cultured chondrocytes containing iPSCs at various ratios. (A) MALDI-TOF MS spectra of GSL-glycans in chondrocytes and iPSCs under co-culture conditions. (B) Linear dynamic ranges of the quantification of $(Hex)_3$, $(Hex)_3(HexNAc)_1(Fuc)_1$, $(Hex)_4(HexNAc)_1$, and $(Hex)_4(HexNAc)_1(NeuAc)_1$ at the various iPSCs containing ratios (iPSCs 20%, 10%, 2.5%, and 1%). Error bars indicate standard deviation (SD) for triplicate measurement.

Supplementary Figure S8

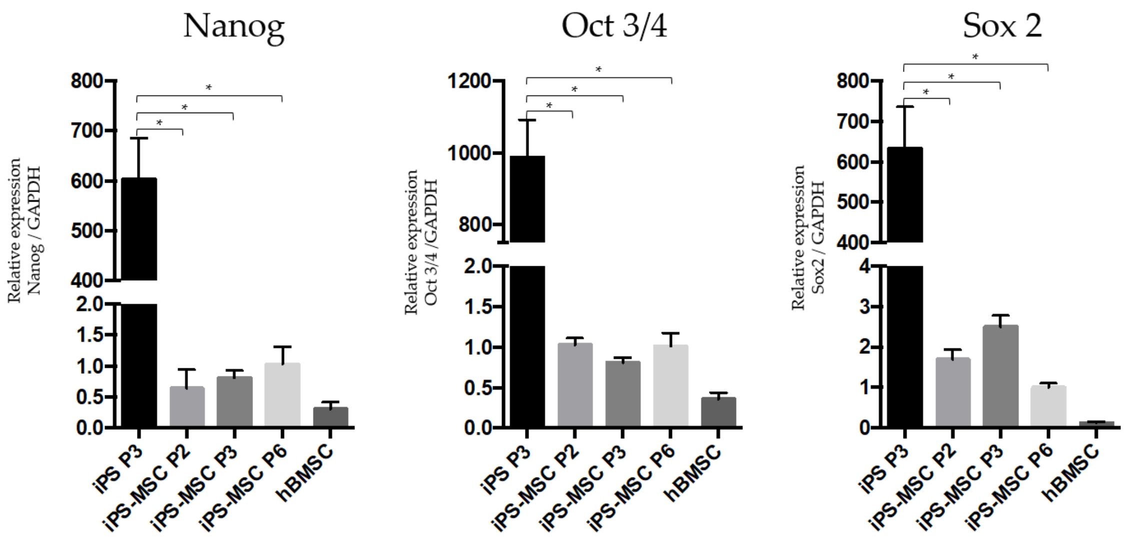

Figure S8. **Gene expression analyses by q-PCR showed significant suppression of pluripotent markers (Nanog, Oct 3/4 and Sox 2) in both of iPS-MSCs P2, P3 and P6.** Human mesenchymal stem cells (hBMCs, purchased from Lonza, Basel, Switzerland) was used as control. Data are presented as mean ± SD. Asterisk denotes statistical significance at *P <0.001

Supplementary Table 1

Gene	Forward primer	Reverse primer
GAPDH	AATTCCATGGCACCGTCAAG	AGGGATCTCGCTCCTGGAAG
Nanog	CAGTCTGGACACTGGCTGAA	CTCGCTGATTAGGCTCCAAC
Oct 3/4	TGTACTCCTCGGTCCCTTTC	TCCAGGTTTTCTTTCCCTAGC
Sox 2	TTCACATGTCCCAGCACTACCAGA	TCACATGTGTGAGAGGGGCAGTGTGC

Supplementary Figure S9

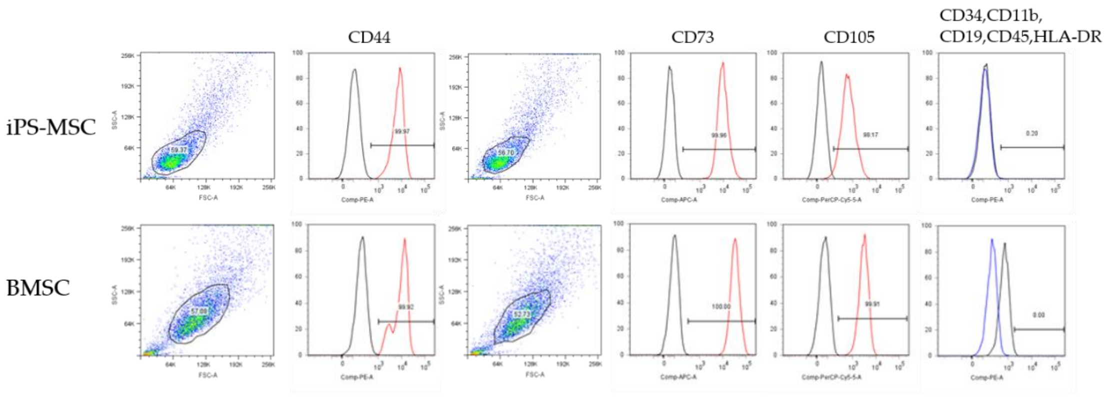

Figure S9. **Flow cytometry analysis of MSC positive and negative markers of iPS-MSC and BMCS.** The results of flow cytometry showed almost all cells were positive for positive markers of MSC (CD 44, 73, and 105) and negative for negative markers of MCS (CD 34, 11b, 19, 45 and HLA-DR) in iPS-MSC P6. iPSC–MSC-like cells (passage 6) and hBMSCs were grown to confluence, harvested by 0.25% trypsin/EDTA, washed with PBS, and resuspended in staining solution consisting of 2% FBS and 25 mM HEPES in PBS. Cell suspensions ($1x10^6$ cells) were mixed with PE mouse anti-human CD44 , APC mouse anti-human CD73, PerCP-Cy™5.5 mouse anti-human CD105 (BD Biosciences, New Jersey, USA), and negative MSC cocktail (PE CD34, PE CD11b, PE CD19, PE CD45 and PE HLA-DR, BD Biosciences). Samples were run on a BD FACSCANTO II instrument (BD Biosciences). Data was analyzed using FloJo Software (Tree Star, USA.).
